# Clinical Features of NMDAR Ab-mediated Encephalitis

**DOI:** 10.15844/pedneurbriefs-29-6-7

**Published:** 2015-06

**Authors:** J. Gordon Millichap, John J. Millichap

**Affiliations:** 1 Division of Neurology, Ann & Robert H. Lurie Children's Hospital of Chicago, Chicago, IL; 2 Departments of Pediatrics and Neurology, Northwestern University Feinberg School of Medicine, Chicago, IL

**Keywords:** NMDAR Ab, Encephalitis, Steroids, Immunoglobulin, Plasma Exchange

## Abstract

Investigators from University of Oxford, Birmingham Children's Hospital, Guy's and St Thomas’, London, UK, performed a prospective surveillance study in children with NMDAR-Ab-mediated neurological disease reported from Nov 2010 to Dec 2011.

Investigators from University of Oxford, Birmingham Children's Hospital, Guy's and St Thomas’, London, UK, performed a prospective surveillance study in children with NMDAR-Ab-mediated neurological disease reported from Nov 2010 to Dec 2011. Over the study period (13 months) 1526 email responses were received from 171 clinicians reporting 33 known and 10 new cases. The incidence was calculated at 0.85/million children/year. The majority of patients were female (23/31, 74% with a median age of 8 years (range 22 months-17 years). Male patients were older (median 11 years, range 6-17 years, p = 0.03). None had a family history of autoimmune disease. Behavioral change and neuropsychiatric features were present in 90% of the 31 children who fulfilled selection criteria. Seizures and movement disorders each occurred in 67%. EEG was encephalopathic in 28/30 (93%); extreme delta brush was not recognized. CSF showed pleocytosis in 45%; 67% had oligoclonal bands. Antibodies detected included antistreptolysin O, IgM Epstein-Barr, antinuclear, antibasal ganglia, and voltage-gated potassium channel-complex. MRI was normal in 65% and showed high signal changes in cortical and subcortical areas in 23%. Typical NMDAR-Ab encephalitis was reported in 24 children and a partial phenotype without encephalopathy in 7. Autonomic features included cardiac arrest in one and hypoventilation in 3. Steroids were prescribed for all patients, 22 (71%) received IV immunoglobulin, 9 (29%) received plasma exchange, and 10 (32%) received second-line immunotherapy. Of 23 diagnosed early, 18 (78%) made a complete recovery compared with only 1 of 8 (13%) diagnosed late (p = 0.002). Seven patients relapsed with 4 needing additional second-line immunotherapy. One (1 of 31, 3%) patient diagnosed late, after 6 months, responded to initial immunotherapy and was subsequently diagnosed and treated for ovarian teratoma with no relapse. [1]

COMMENTARY. Pediatric NMDAR-Ab encephalitis often presents with neurological rather than psychiatric symptoms, the more common presentation in adults with the disease. Patients diagnosed and treated early with immunotherapy have a better prognosis than those treated late [1]. Anti-NMDAR Abs in CSF is the diagnostic marker for NMDAR encephalitis, and the diagnosis may be supported by the EEG pattern “extreme delta brush.”[2]. This pattern named because of its resemblance to the EEG of premature infants is reported in 30% of adult patients with NMDAR encephalitis. Extreme delta brush is associated with more severe neurological/behavioral symptoms, prolonged hospitalization and increased days of cEEG monitoring.

In pediatric patients with behavioral disorders and abnormal movements, early EEG patterns may be suggestive of anti-NMDAR encephalitis. Consecutive polygraphic video-EEG recordings in 9 children with NMDAR encephalitis were analyzed and in 6 patents, the waking EEG showed preserved background activity and either focal or unilateral hemispheric slowing. The outcome with focal EEG slowing was more favorable than in the 3 children with diffuse slowing. Unilateral abnormal movements contralateral to the hemispheric slowing were also indicative of milder clinical severity when compared with generalized abnormal movements and diffuse slowing [3].
